# Dogs on the Move: Factors Impacting Animal Shelter and Rescue Organizations’ Decisions to Accept Dogs from Distant Locations

**DOI:** 10.3390/ani6020011

**Published:** 2016-02-03

**Authors:** Kaitlyn E. Simmons, Christy L. Hoffman

**Affiliations:** Department of Animal Behavior, Ecology, and Conservation, Canisius College, 2001 Main Street, Buffalo, NY 14208, USA; simmonsk@canisius.edu

**Keywords:** animal shelter, transfer, transport, relocation, domestic dog, animal welfare

## Abstract

Long-distance dog transfer programs are a topic of burgeoning interest in the animal welfare community, but little research has focused on such programs. This exploratory study, which surveyed 193 individuals associated with animal shelter and rescue organizations in the United States, evaluated factors that impacted organizations’ decisions to transfer in dogs over long distances (>100 miles) and assessed what criteria were commonly valued by destination organizations. Specifically, we examined the following aspects of long-distance transfer programs: (1) logistics of long-distance dog transfers; (2) factors impacting dog selection; (3) medical requirements; (4) partnerships formed between source and destination organizations; and (5) perceptions of long-distance dog transfer programs by individuals affiliated with the destination organizations. This study revealed that many logistical considerations factor into transfer decisions and the formation of healthy partnerships between source and destination organizations. Participants indicated their organization’s willingness to receive dogs of various sizes, coat colors and ages, but organizations often had restrictions regarding the breeds they would accept. Study findings indicate some organizations have strict quarantine policies and pre-transfer medical requirements, while others have no such requirements.

## 1. Introduction

Companion animals are a common part of American life, with 36.5% of American households owning dogs [1]. However, statistics regarding the number of companion animals in shelters in the United States reveal a vast problem. According to the Association for the Prevention of Cruelty to Animals [2], 3.9 million dogs enter shelters in the United States annually, and 1.2 million are euthanized. Approximately 35% of dogs entering shelters are adopted; 31% are euthanized; and 26% are returned to their owners [2]. 

Animal transfer programs provide one potential way to improve outcomes for shelter dogs, particularly since there are regional differences in pet ownership patterns. For instance, the percentage of households owning a dog varies by state and ranges from 24% in Massachusetts to 48% in Arkansas [1]. Furthermore, data from studies examining adopter preferences suggest there may be regional differences in preferences regarding the age, size, color and breed of dogs [3,4,5,6]. As dog ownership patterns fluctuate vastly between states and across regions, information on the popularity of dogs and preferred characteristics of dogs by state and region can be crucial to developing ways to raise adoption rates and lower euthanasia rates through transfer programs. 

Despite vast interest in the subject of animal transfer programs in the animal welfare community [7], much of the debate regarding the merits of such programs is based on anecdotal evidence. A perception held by some is that puppies and small dogs are the only dogs that shelters and rescue groups will accept through transfer programs, and there is concern that animal transfer programs may result in fewer dogs from the destination organization’s community getting adopted [8]. Other concerns include risk of disease transmission and the introduction of diseases into new geographic areas [9,10]. 

Supporters of animal transfer programs contend there are many benefits associated with animal transfers for both dogs and animal shelter and rescue groups and argue that health protocols can quell the risk of disease transmission [11]. In addition, relocating animals from areas with high animal populations and limited demand to areas with high demand and fewer homeless dogs may improve the live release rates of shelter dogs [10]. Furthermore, dog transfer programs can increase the variety of dogs available for adoption, which is something prospective dog adopters value [12], and can thereby provide communities with adoptable dogs that people previously may have purchased from a breeder or pet shop. Such programs may also allow shelters with limited resources to transfer animals to organizations that have more medical and financial capabilities [10].

The present study investigated what factors are most influential in destination organizations’ decisions to transfer in dogs over distances greater than 100 miles. Specifically, this exploratory study examined five aspects of long-distance dog transfer programs: (1) logistics of long-distance dog transfers; (2) factors impacting dog selection; (3) medical requirements; (4) partnerships formed between source and destination organizations; and (5) perceptions of long-distance dog transfer programs by individuals on the receiving side. In addition, we assessed whether the region in which the destination organization was located, the size of the organization and whether it had a facility influenced transfer-related decisions and policies. Uncovering common themes associated with organizations’ decisions to select particular dogs for long-distance transfer could contribute to a better understanding of the myriad of factors at play within this complex situation. 

## 2. Methods

### 2.1. Participant Recruitment

We distributed the survey to shelter and rescue organizations across all 50 states within the United States. The survey was open to any shelter or rescue staff and volunteers who were 18 years of age and older and were directly involved in deciding which dogs to transfer into their organization from distances greater than 100 miles. The first question of the survey asked if participants had knowledge of anyone else in their organization completing the survey. Individuals whose response to this question was “yes” were disqualified from participating in the rest of the survey. To maintain confidentiality and encourage candid responses, we did not ask participants for their names or their organizations’ names. We recruited the sample population using an e-mail list that we generated primarily from searching the Petfinder website. The list included approximately 3000 United States shelters. We e-mailed survey invitations to a variety of organizations, including private organizations, government-run animal control facilities, rescue groups and sanctuaries. Fifteen percent of the e-mails bounced back as undeliverable. Of the e-mails that were delivered, we do not know how many reached organizations that engaged in long-distance transfers of dogs. Prospective participants did not receive follow-up or reminder e-mails regarding the survey. Two-hundred sixty-two individuals started the survey. Two-hundred eighteen of those met the survey’s eligibility requirements, and 193 completed the entire survey, yielding an 89% completion rate by eligible participants. 

### 2.2. Measures and Analysis

Participants completed a 5-part survey hosted by the website SurveyMonkey. The survey, which has been included as a supplement to this paper, contained multiple choice and open-ended questions. The first section asked questions about participant age, sex and role within their organization and also asked participants to indicate which state their organization was located in, to report whether their organization was located in an urban, rural or suburban environment and to specify whether their organization was classified as a shelter, rescue group, sanctuary or some combination of these things.

The second section of the survey asked about the logistics of the organization’s long-distance transfer program. Questions focused on organizational resources, including number of paid staff members, whether the organization had a physical location, number of dogs transferred from long distances, funding and what resources were allocated for transfer. Following this, a third section explored the organization’s quarantine policy and pre-transfer medical requirements. The fourth section delved into factors impacting the organization’s decisions to select particular dogs. For instance, the survey asked participants how much dog size, age, breed, behavior and origin impacted transfer decisions. Because we were doubtful participants would have detailed intake records from their organization readily available when completing the survey, many of the questions were phrased in a hypothetical manner (e.g., how likely would your organization be to transport in dogs of the following sizes?). The final section of the survey inquired about participants’ personal opinions regarding dog transfer programs. 

Some multiple choice questions allowed respondents to select other as an answer choice and provide an open-ended response to the question. The open-ended responses were, when applicable, incorporated into one or more of the answer choices, or new categories were created for the responses. Of particular note, if respondents answered “yes” to the question regarding whether their organization required certain medical treatments prior to transfer, they were directed to an open-ended text box asking them to describe the medical treatments that were required.

The survey was designed with the intent of requiring each participant to provide an answer to each question, but a few errors in the survey setup resulted in missing responses for six questions. Three participants did not indicate how many dogs their organization takes in each year (Supplementary files Q13), and 10 did not indicate how many dogs they receive from long-distance transfer programs (Supplementary files Q18). Forty-three of the 47 participants who indicated that their organizations did not require the dogs they received to undergo certain medical treatments prior to intake did not rate how factors such as bite history, dogs’ photographs, breed, coat color, shelter space, funding and potential for disease transmission influenced their decisions to take in particular dogs (Supplementary files Q30 and 31). These 43 participants also were not asked whether they had preferences for purebred or mixed-breed dogs (Supplementary files Q32) or how likely they would be to accept dogs of various sizes (Supplementary files Q33). 

Given the descriptive nature of this study, analyses were comprised of frequency calculations and chi square tests. Specifically, we ran chi square tests to determine whether region, whether an organization had a facility and the number of dogs per year an organization received from long-distance transfers had any bearing on transport-related logistics, whether organizations had restrictions on the breeds they accepted and whether organizations had quarantine and/or pre-transport medical requirements. In addition, we used a chi square test to see if there was a relationship between organizations’ tendencies to have quarantine and pre-transport medical requirements. Statistical analyses were completed using IBM SPSS 22.0 (Chicago, IL, USA).

## 3. Results

### 3.1. Characteristics of Survey Participants and Their Organizations

Most survey participants were female, 35 years or older and represented animal rescues that were private and had no government contracts. Participants came from 41 states and represented all regions of the United States and all types of communities (*i.e.*, urban, suburban and rural). Table 1 provides details about the sample.

**Table 1:** Sample description.

Factors	Count (%)
Sex of Participants	
Female	185 (95.9%)
Male	8 (4.1%)
Age of Participants (years)	
18–24	6 (3.1%)
25–34	29 (15.0%)
35–44	41 (21.2%)
45–54	64 (33.2%)
55–64	39 (20.2%)
65+	14 (7.3%)
Regions	
Northeast	78 (40.4%)
Midwest	52 (26.9%)
West	33 (17.1%)
South	30 (15.5%)
Community Type	
Rural	64 (33.2%)
Suburban	63 (32.6%)
Urban	30 (15.5%)
Spans Multiple Communities	36 (18.7%)
Type of Organization	
Rescue	140 (72.5%)
Shelter	40 (20.7%)
Sanctuary or Combination	13 (6.7%)
Government Involvement	
Private, No Government Contract	138 (71.5%)
Private, Government Contract	21 (10.9%)
Publicly-Funded Municipal Agency	1 (0.5%)
Other	33 (17.1%)

The majority of participants (69%, *n* = 134) operated at a volunteer-run organization. Eighty-four percent (*n* = 162) worked for an organization that placed limits on the numbers and/or types of dogs it accepted (*i.e.*, selective or limited admission), and 16% (*n* = 31) worked for an organization that accepted all dogs surrendered to it by the public (*i.e.*, open intake). Twenty-two percent (*n* = 42) of participants stated their organization focused on helping dogs of particular breeds or sizes.

Organizations represented in survey responses varied greatly regarding the annual total number of dogs they took in and the number of dogs they brought in from distances greater than 100 miles (Table 2). Respondents identified a wide range of facilities and locations from which their organizations operated. Thirty-one percent (*n* = 60) of the organizations represented had a stand-alone building that functioned exclusively for the organization’s purposes. Sixty-five percent (*n* = 126) reported that their organization operated out of a home or a network of foster homes. Five percent (*n* = 9) reported housing at least some of their dogs at veterinary clinics, boarding facilities or grooming businesses, and 2% (*n* = 3) reported operating out of prison facilities.

**Table 2:** The number of organizations belonging to each “number of dogs” category according to their total annual intakes of dogs and their total annual intakes of dogs from long-distance transfers.

Number of Dogs	Total Intakes	Intakes from Long-Distance Transfers
Fewer than 10	7 (3.6%)	23 (11.9%)
10–49	20 (10.4%)	51 (26.4%)
50–99	40 (20.7%)	38 (19.7%)
100 or more	123 (63.7%)	71 (36.8%)
Missing Data	3 (1.6%)	10 (5.2%)

### 3.2. Logistics

Participants reported having to consider a number of logistical details, including funding, foster home or shelter space and arrangement of transportation. Forty-three percent (*n* = 82) stated their organization was responsible for funding the transfer; 9% (*n* = 17) relied solely on volunteers and independent donors; 19% (*n* = 36) indicated the source organization was responsible; 8% (*n* = 15) reported the source and destination organizations shared the cost; and 22% (*n* = 43) explained the funding source depended on the situation, transport mode and availability of funding. Organizations in the South and Northeast tended to be more likely than those in the Midwest and West to completely cover the transfer costs, χ^2^(3) = 7.71, *n* = 193, *p* = 0.052. The number of dogs organizations brought in annually from long-distance transfers had no effect on their organization’s likelihood of funding transfers, χ^2^(3) = 2.68, *n* = 183, *p* = 0.44. Neither did having a facility, χ^2^(1) = 2.00, *n* = 193, *p* = 0.16.

The actual transfer of dogs involved numerous people. For transfer-related questions, survey participants were allowed to select all answer choices that were relevant to their organization. The most common modes of transport reported by participants were by car, van or truck (96%, *n* = 185) and airplane (34%, *n* = 65). Fifty-six percent (*n* = 108) reported that staff or volunteers from their organization were involved in transporting at least some of the dogs; 41% (*n* = 79) said staff or volunteers from the source organization were involved; and 53% (*n* = 103) said transport groups that operated independently of the source and destination organizations were involved. There was no effect of region, the number of dogs organizations transferred in annually or whether an organization had a facility on whether anyone from the destination organization participated in the transport (region: χ^2^(3) = 5.05, *n* = 193, *p* = 0.17; number transferred: χ^2^(3) = 0.02, *n* = 183, *p* = 0.999; facility: χ^2^(1) = 0.20, *n* = 193, *p* = 0.67). Similarly, these variables did not impact whether the organizations relied on the source organization to conduct the transport (region: χ^2^(3) = 1.06, *n* = 193, *p* = 0.79; number transferred: χ^2^(3) = 0.39, *n* = 183, *p* = 0.94; facility: χ^2^(1) = 1.18, *n* = 193, *p* = 0.28). Compared to other regions, there was a tendency for organizations in the Northeast to be more likely to use independent organizations for transports, χ^2^(3) = 7.69, *n* = 193, *p* = 0.053. Organizations that transferred in more than 50 dogs per year were more likely to use independent transport organizations than were the organizations that brought in fewer dogs, χ^2^(3) = 12.83, *n* = 183, *p* = 0.005. Whether the destination organizations had a facility had no bearing on whether they used independent transport organizations, χ^2^(1) = 1.57, *n* = 193, *p* = 0.21. 

### 3.3. Expectations Regarding Dogs’ Physical Characteristics, Origin and Adoptability

Destination organizations tended to place importance on certain characteristics of dogs when deciding which ones to transfer. Due to a survey design error, only 152 of the 193 participants answered most of the questions described in this section. Ninety percent (*n* = 137) of the 152 participants said descriptions were important, and 78% (*n* = 118) said photographs were important. Thirteen percent (*n* = 19) reported their organization’s decisions were impacted by stories that evoke media attention or stir public sentiment. 

**Figure 1:**
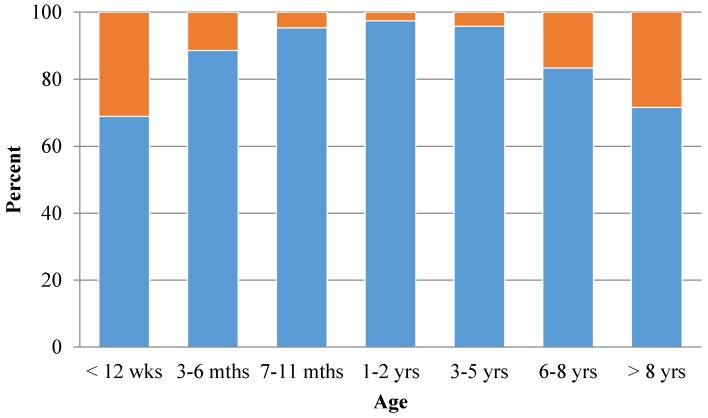
Likelihood of organizations to accept dogs of different ages as part of long-distance transfers. Blue bars represent likely to accept, and orange bars represent unlikely to accept.

A large percentage of participants indicated that a dog’s breed, age and size influenced their transfer decisions, but coat color and sex typically did not. Sixty-two percent (*n* = 94) rated dog breed as important, and age and size were ranked as important by 46% (*n* = 70) and 47% (*n* = 71), respectively. For each size category, which ranged from under 10 lbs–over 90 lbs, 68% or more of participants said their organizations would be likely to transfer in dogs of that size. Regarding responses about an organization’s willingness to transfer in dogs of particular ages, dogs aged 3 months–8 years old were all rated as likely to be accepted by over 83% of respondents (Figure 1). Approximately 70% of participants reported their organizations would be willing to accept dogs 12 weeks and younger or those older than eight years. Only 7% (*n* = 11) deemed coat color and sex of the dog to be important. 

When participants were asked if their organization would decline to transfer in dogs of certain breeds from long distances, 54% (*n* = 104) answered yes. Participants in the Midwest were the most likely to state there were breeds their organization would decline, and participants in the West were the least likely, χ^2^(3) = 8.53, *n* = 193, *p* = 0.04. The number of dogs transferred in annually and whether an organization had dedicated facilities had no bearing on organizations’ tendencies to restrict breeds (number transferred: χ^2^(3) = 2.69, *n* = 183, *p* = 0.44; facility: χ^2^(1) = 1.08, *n* = 193, *p* = 0.30). 

Of the participants whose organizations had restrictions on breeds accepted, 40% (*n* = 42) stated that their organization was focused on dogs of particular breeds or sizes. Organizations that were not breed- or size-specific, but had breed-related restrictions commonly cited an unwillingness to accept “bully breeds,” “pit bulls” or “pit mixes” from long distances. Some organizations also expressed a reluctance to accept Akitas, German shepherds, Rottweilers, Doberman Pinschers and Chow Chows. Reasons given for not accepting these breeds included insurance restrictions, breed-specific legislation (BSL), concerns about potential behavioral issues and/or lack of adopter interest. Furthermore, some participants noted that these types of dogs were prevalent in their local shelters and that they chose to pull them locally as opposed to bringing them in from long distances. In addition, hounds, black Labradors and Chihuahuas were mentioned by some respondents as dogs their organizations would not accept. The reasons participants gave for not accepting these dogs were that there was already an abundance of them in their community or that there was a lack of interest in these breeds among local adopters. Some participants explicitly stated they focused on transferring in breeds that were rare in their communities. 

Participants indicated that the safety of both the dog and those interacting with the dog were factors commonly considered when making transfer-related decisions. Eighty-five percent (*n* = 129) reported that a motivating force behind selecting a particular dog was the likelihood that the dog would be euthanized at its current location. Ninety-five percent (*n* = 145) rated a dog’s bite history and 91% (*n* = 139) rated a dog’s potential for transmitting disease as important factors influencing their decision not to transfer a particular dog. 

**Figure 2:**
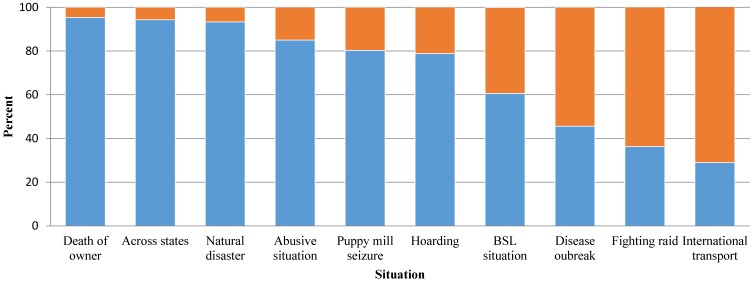
Likelihood of organizations to transfer in dogs in need of rehoming due to various circumstances. Blue bars represent likely, and orange bars represent unlikely. BSL, breed-specific legislation.

All 193 participants were provided with a number of circumstances that might result in the need for dogs to be transferred, and we asked them how likely their organization would be to accept dogs from each situation. For example, we asked how likely they would be to accept dogs from hoarding situations, puppy mill seizures, international transports and natural disasters. Figure 2 depicts the percentages of participants who stated their organizations would be willing to transfer in dogs under various circumstances. Notably, organizations tended to be reluctant to receive animals from international locales and dog fighting raids, but over 90% of participants reported a willingness to accept animals displaced by natural disasters, left homeless after their owner died or from other states. Forty-six percent (*n* = 88) were willing to accept dogs from shelters experiencing disease outbreaks.

### 3.4. Medical Requirements

Medical requirements varied greatly among destination organizations. Sixty-four percent of respondents (*n* = 124) stated their organizations required that all dogs coming to them from long distances go through a mandatory quarantine period. Of the organizations that had a quarantine period, 72% (*n* = 89) stated their organization carried out the quarantine; 9% (*n* = 11) indicated that both the source and destination organizations shared the responsibility; 15% (*n* = 19) stated that the source organization was responsible; and 4% (*n* = 5) selected other. Those who selected other tended to report that their quarantine protocol was situation dependent. The length of the quarantine period varied widely across organizations, with respondents most commonly selecting 2–9 days (37%, *n* = 46) or 10–14 days (52%, *n* = 65). Seven percent (*n* = 9) of respondents said their organizations quarantined for over 14 days. Three percent (*n* = 4) did not provide a specific length and instead indicated the length was situation dependent. Four participants explicitly stated the quarantine period length was mandated by state and/or local regulations, and five indicated that their requirements differed for puppies and adult dogs, with puppies having longer quarantine periods. 

Seventy-six percent (*n* = 146) of participants’ organizations required that dogs undergo certain medical treatments prior to transfer. Compared to respondents who reported their organizations had quarantine policies, those who did not were more likely to report that their organizations did not have any pre-transfer medical requirements, χ^2^(1) = 10.36, *p* = 0.001. Thirteen percent (*n* = 26) of participants stated their organizations had neither quarantine policies nor pre-transfer medical requirements for dogs received from long-distance transfers. 

A couple factors were associated with organizations’ tendencies to institute quarantines and pre-transfer medical requirements. Organizations in the Northeast were more likely to require quarantines and have pre-transfer medical requirements than organizations in other regions (quarantine: χ^2^(3) = 18.34, *n* = 193, *p* < 0.001; medical requirements: χ^2^(3) = 19.03, *n* = 193, *p* < 0.001). There was no relationship between the number of dogs an organization transferred in from long distances and whether the organization had quarantine requirements, χ^2^(3) = 5.94, *n* = 183, *p* = 0.11; however, organizations that transferred in more than 50 dogs per year were more likely than smaller organizations to have pre-transfer medical requirements, χ^2^(3) = 15.51, *n* = 183, *p* = 0.001. Whether organizations had their own designated facilities, however, had no bearing on their likelihood of having quarantine policies or pre-transfer medical requirements (quarantine: χ^2^(1) = 1.25, *n* = 193, *p* = 0.26; medical requirements: χ^2^(1) = 0.05, *n* = 193, *p* = 0.83).

The medical treatments organizations required pre-transfer varied extensively, with some requiring that the majority of medical treatments take place prior to transfer and others requiring minimal or no medical treatments. Nineteen percent (*n* = 36) mentioned the dogs they received needed to have health certificates, which are issued by veterinarians. Fifty-eight percent (*n* = 111) required rabies vaccines, and 54% (*n* = 104) required distemper/parvovirus combination vaccines. Thirty-five percent (*n* = 68) required dogs receive heartworm testing, treatment and/or preventative treatment prior to transfer, and 6% (*n* = 11) required testing for tick-borne illnesses, such as Lyme disease, ehrlichiosis and anaplasmosis. Table 3 provides additional details regarding medical requirements, including the percentage of participants in each region whose organizations required each procedure.

**Table 3:** The number of participants within each region and overall who stated their organizations had pre-transfer medical requirements (percentages in parentheses).

Procedure	Midwest	Northeast	South	West	Total
Rabies Vaccine	28/52 (53.8%)	55/78 (70.5%)	13/30 (43.3%)	15/33 (45.5%)	111 (57.5%)
Distemper/Parvovirus Vaccine	20/52 (38.5%)	54/78 (69.2%)	13/30 (43.3%)	17/33 (51.5%)	104 (53.9%)
Heartworm Test, Treatment, and/or Preventative	11/52 (21.2%)	38/78 (48.7%)	9/30 (30.0%)	10/33 (30.3%)	68 (35.2%)
Bordetella Vaccine	11/52 (21.2%)	23/78 (29.5%)	9/30 (30.0%)	8/33 (24.2%)	51 (26.4%)
Fecal Testing/Deworming	9/52 (17.3%)	25/78 (32.1%)	6/30 (20.0%)	6/33 (18.2%)	46 (23.8%)
Spay/Neuter	6/52 (11.5%)	24/78 (30.8%)	8/30 (26.7%)	5/33 (15.2%)	43 (22.3%)
Testing and/or Treatment for External Parasites	6/52 (11.5%)	15/78 (19.2%)	2/30 (6.7%)	5/33 (15.2%)	28 (14.5%)
Testing and/or Treatment for Tick-Borne Illnesses	0/52 (0.0%)	9/78 (11.5%)	2/30 (6.7%)	0/33 (0.0%)	11 (5.7%)
Microchip	0/52 (0.0%)	6/78 (7.7%)	2/30 (6.7%)	1/33 (3.0%)	9 (4.7%)

### 3.5. Partnerships

Participants provided details regarding their organization’s relationships with the organizations from which they received dogs. Sixty percent (*n* = 115) reported having a relationship with 1–5 organizations; 20% (*n* = 38) worked with six or more organizations; 11% (*n* = 22) worked with different organizations each time; and 9% (*n* = 18) either did not know how many organizations their group partnered with or stated that their organization tended to take long-distance transfers from individual rescuers. 

Partnerships sometimes failed, and 53% (*n* = 103) of participants indicated their organizations had previously transferred in dogs from groups with which they no longer partnered. There were a variety of reasons for the partnerships dissolving. Some of the common reasons offered included miscommunication between organizations, disorganization, feelings of deception and failure to keep agreements. Twenty-one percent (*n* = 41) of participants believed that a source organization had deliberately deceived them in an effort to relocate a dog. Twenty-seven percent (*n* = 52) believed the source organization did a poor job communicating a dog’s behavioral needs prior to arrival, and 22% (*n* = 43) reported that the source organization did a poor job representing a dog’s medical needs. Only 5% (*n* = 9) indicated that the relationship ended because the source facility did not have dogs that were of interest to the destination organization’s community. Regarding failed partnerships, one respondent explained, “(The source organization’s) transports are unorganized and delayed. When the most recent transport was six hours late and it had been an on-going pattern, I stopped working with them.” Another respondent commented, “The key is having a solid relationship between the sending and receiving groups. If sending groups do not vaccinate properly or (they) knowingly send sick dogs, it will have a negative effect on the receiving shelter and will make receiving groups not want to work with you. Definitely take time to build a solid trusting relationship, and transporting animals can be a win-win for all.” 

Some respondents reported that they tried to form partnerships with organizations that are taking active steps in their local community to address the dog overpopulation problem. One participant stated, “We are located in Illinois but pull most of our dogs from Alabama. It is extremely expensive for us…however, we have built a very strong relationship with the sending shelter and are confident that they are making a difference in their community. We will only work with long-distance shelters if the local community has a plan and purpose to increase spay and neuter programs in their area.” Another respondent recounted that her organization broke off its partnership with a shelter upon finding out that the source organization was adopting out dogs that were not spayed or neutered. 

### 3.6. Participants’ Personal Beliefs Regarding Transfer Programs

When participants were asked whether they personally agreed with, disagreed with or had no opinion about statements regarding long-distance dog transfer, most indicated they held highly positive sentiments. Seventy-seven percent or more agreed or strongly agreed with each of the following statements about transfer programs: they provide communities with dogs of particular breeds, ages or sizes that individuals would otherwise purchase; they positively impact their organization; they can help combat the pet overpopulation problem; and they can help lower euthanasia rates across the nation. Forty-three percent (*n* = 83) believed that dog transfer programs helped raise publicity for their organization. Opinions were mixed regarding whether such programs contribute to the spread of animal diseases, with 30% (*n* = 58) agreeing or strongly agreeing that these programs can lead to the spread of disease, 22% (*n* = 43) indicating they were neutral about this and 48% (*n* = 92) disagreeing or strongly disagreeing. Eight percent or fewer agreed or strongly agreed with the following statements: these programs make it harder to adopt out the dogs destination organizations currently have; they are not worth all the effort and time staff members have to put into the logistical details required; and they are overrated and do not help raise adoption rates or lower euthanasia rates.

## 4. Discussion

This study provides insights into the logistics of long-distance dog transfer programs and what factors matter most to the destination organizations. The data from this study can help elucidate for the animal welfare community how destination organizations determine the groups with which they will partner, how they handle the risks of disease transmission and what factors lead to successful partnerships between organizations. Although the sample was not representative of all organizations engaging in transfer programs, the results illustrate that there is much variation across animal shelter and rescue organizations regarding how they make and execute transfer-related decisions.

Volunteers and donors play key roles on the logistical side of dog transfers. Most organizations represented in this study depended on private donations, and many were either all volunteer-run or relied heavily on volunteer help for their transfer programs. Volunteers provided foster homes, secured funding for transfers, moved dogs from source to destination organizations and coordinated transfers. 

Participants reported that many factors had to be considered when selecting dogs for transfer. They commonly highlighted the importance of knowing the medical and behavioral needs of the dogs prior to arrival. This information enabled them to estimate the time and money they would need to invest in these dogs. If destination organizations felt a partner organization had sent them dogs without fully disclosing their medical or behavioral needs, they were inclined to terminate the relationship. 

Many within the animal welfare community have concerns about the potential of animal transfer programs to contribute to disease transmission [10]. Indeed, long-distance pet movement around Europe has contributed to the spread of vector-borne pathogens, including leishmaniasis and babesiosis [13,14], and following the relocation of dogs from the Gulf Coast region after Hurricane Katrina, heartworm (*Dirofilaria immitis*) and other infectious diseases became more common in communities that received these dogs [15]. In our study, disease transmission was a concern of destination organizations, although 70% of participants either disagreed with or had no opinion about the statement, “I believe dog transfer programs can lead to the spread of animal disease.” Furthermore, 26 participants indicated that their organizations had neither quarantine policies nor pre-transfer medical requirements for dogs received from long-distance transfers. Additionally, we found that organizations that accepted fewer than 50 dogs per year from long-distance transfers and organizations outside the Northeast tended to have fewer pre-transfer medical requirements. 

More than half of participants reported their groups required dogs to have rabies vaccines and distemper/parvovirus combination vaccines prior to transfer, but overall, there was a high degree of variation regarding pre-transfer medical requirements. Only 19% of participants mentioned requiring health certificates, even though most state laws require them for dogs entering their state. As most of the dogs were transported by car, it is important to note that pre-transfer medical requirements, or lack thereof, have the potential to impact not only the destination organizations and their communities, but also the states and regions through which the dogs travel.

Respondents commonly reported that destination organizations wanted to be actively involved in selecting the dogs for transfer. Nearly all respondents desired a description of the dogs and believed it was important to know whether a dog had a bite history. Most participants also placed a high priority on being able to see a photograph of the dog. Respondents may believe that a photograph signals transparency on the part of the sender or allows the destination organization to draw its own conclusions about a dog’s breed, color or size. In addition, individuals selecting dogs for transfer may be attracted to dogs whose photos have certain characteristics. This is certainly the case for dogs adopters; dogs that are photographed outdoors or that are looking directly at the camera tend to be adopted more quickly [16].

The sex and coat color of the dogs the organizations received were of little import. Furthermore, although nearly half of participants rated age and size as important factors influencing their decisions to select particular dogs, organizations were willing to take in dogs of various ages and sizes. Although respondents were less likely to transfer in dogs under 12 weeks old or over eight years old than dogs between three months and eight years, the majority stated they would still be willing to transfer in dogs from these age groups. The motives and procedures for moving young puppies and senior dogs merit more exploration because there are additional medical, behavioral and welfare concerns associated with transporting these dogs.

Breed played a key role in many destination organizations’ decisions, which may be explained by the inclusion of numerous breed-specific rescue groups in the current study and by groups’ desires to diversify their shelter or rescue’s population. By introducing new breeds into the organization’s population, transfer programs may succeed in creating the variety of options prospective adopters have been shown to desire [12]. Numerous organizations in our study would not transfer in bully breeds. Some reasons participants mentioned for not accepting bully breeds were overpopulation of bully breeds in the destination organization’s area, concern regarding behaviors associated with these breeds, regulations in the destination area regarding bully breeds and a lack of adopter interest in bully breeds. Thus, communities with a surplus of dogs might do well to focus their efforts on promoting non-bully breeds for long-distance transfers while working to increase adoptions of bully breeds within their communities. 

An area of concern regarding dog transfer has been the fear that if organizations brought in dogs from long distances, then local dogs would be overlooked by the public and suffer [8]. When participants were asked for their personal opinions about this, 77% disagreed that bringing in dogs from long distances made it harder to adopt out local dogs from their organization, and 77% felt that long-distance transfers allowed their organization to provide their community with dogs that adopters would otherwise purchase. Additional investigation is needed to determine if participants’ beliefs are supported by data from destination organizations.

Although some news articles and bloggers have expressed concern that organizations are motivated to bring in dogs from long distances for publicity purposes, for monetary gain and/or to obtain puppies or toy breeds [8,17], this did not seem to be the case for the organizations represented in this study. Some organizations did limit their intake to certain breeds, but organizations seemed willing, on the whole, to accept a variety of dogs requiring varying degrees of investment. In addition, while 43% of participants felt their organization’s dog transfer program helped raise publicity for the organization, only 13% stated they selected individual dogs based on their ability to evoke media attention through their story. It must be noted, however, that while our data do not support the concern that destination organizations make choices purely for their own benefit, participants were not required to provide detailed records of the dogs they had actually received. Thus, it is possible some participants were providing what they felt were the most socially-acceptable answers to questions regarding motivating factors driving dog selection. 

The majority of participants expressed the belief that long-distance transfer programs, despite their associated financial and logistical challenges, could help lower euthanasia rates across the nation and combat the pet overpopulation problem. This belief is shared by many supporters of long-distance transfer programs [10,11], although data are needed to quantify the actual impact of such programs on the pet overpopulation problem. If positive outcomes are attained through long-distance transfer programs, animal shelter staff and volunteers stand to benefit, as well as dogs, given the emotional toll euthanasia of companion animals takes on animal care workers [18,19]. 

### Strengths, Limitations and Future Directions

There is a lack of existing data on dog transfer programs, and this exploratory study served as an initial step to understanding important factors regarding long-distance dog transfer programs. Because there is no database that identifies animal shelter and rescue organizations that receive dogs from distances exceeding 100 miles, we do not know how many of our survey invitations reached groups that serve as destination organizations for long-distance dog transfer programs. Due to this, we could not calculate a response rate. Nevertheless, study participants from across the United States completed the survey and represented a range of viewpoints regarding the intake of dogs that are sourced from distances exceeding 100 miles. As participation in this study was limited to individuals affiliated with organizations engaged in long-distance transfers, our findings cannot be extrapolated to individuals working in organizations that do not have such programs. Surveying these individuals would provide helpful information regarding why some organizations do not participate in these programs. While this study does provide insights into factors involved in long-distance dog transfers, it is possible and likely that transfers of dogs over short distances are influenced by very different factors. Thus, there is a need for a separate study focused on community-based transfer programs, such as transfers that occur between municipal shelters and other local shelters or rescue groups. 

This study was limited to exploring perceptions and requirements of destination organizations, but it would be helpful for future studies to collect data from the perspective of source organizations and individuals who are responsible for driving or flying the dogs. Developing an understanding of concerns and considerations expressed by both source and destination organizations could help strengthen the communication and understanding between the two sides of long-distance dog transfer programs. A study of the transporters, who often operated independently from the larger organizations represented in our study, would provide insight into efforts made to minimize the potential negative impacts of transport on dog welfare and would draw attention to transport-related welfare concerns that merit consideration. Welfare issues associated with long-distance transfer are an area of particular concern given that transport can adversely affect dog behavior and has the potential to create new behavior problems (e.g., phobias) [20].

Due to the exploratory nature of this study, we were unable to address all considerations involved in long-distance transfers. For instance, data were not broken down based on distance of transfers or whether transfers crossed state lines or international boundaries. These are areas needing additional investigation, because average distance traveled, time spent traveling and state or international boundaries crossed can impact transfer-related expenses, animal welfare and animal health. More work is also needed to examine what photographic or descriptive features are of most interest to destination organizations and to determine how the geographic locations of source organizations factor into transfer decisions. In addition, future studies might examine the finding that nearly half of participants expressed a willingness to accept dogs from shelters experiencing disease outbreaks, even though participants commonly conveyed a desire to minimize risks of disease transmission. 

Finally, this study provided a variety of insights into many aspects of long-distance dog transfer programs, but did not examine how long-distance transfers impact shelter and rescue statistics, such as average length of stay, live release rate and disease prevalence. Future studies should explore how these programs impact outcomes, not only for the transferred dogs, but also for the dogs that originate within the destination shelters’ communities and the dogs remaining in the source organizations. 

## 5. Conclusions

This study revealed numerous factors that can impact the success of long-distance dog transfers and many logistical considerations that factor into transfer-related decisions. The planning and funding for these programs often depend on volunteers’ efforts. Our findings suggest that destination organizations tend to be willing to receive dogs of various sizes, coat colors and ages. Many organizations, however, are reluctant to accept some breeds, largely due to the desire to diversify the destination organization’s population, to organizations having a breed-specific focus or to BSL. There is a great deal of variation across organizations regarding quarantine policies and pre-transfer medical requirements. Given the potential of transfer programs to hasten the spread of infectious diseases and adversely affect animal welfare, it is imperative that destination organizations consult with veterinarians and established animal transport guidelines, such as those published by the Association of Shelter Veterinarians [21], to ensure programs do not cause disease outbreaks or other welfare problems. Although the risks associated with transfer programs must be considered, the vast majority of survey participants viewed long-distance transfer programs favorably and as having the potential to reduce problems associated with pet overpopulation. With over one million dogs euthanized each year [2] and a desire within the animal welfare community to reduce this number, this study provides important insights into factors that facilitate the formation of successful transfer programs and partnerships between source and destination organizations. 

## Supplementary Files

Supplementary File 1
