# Supplementary material for: Dogs on the Move: Factors Impacting Animal Shelter and Rescue Organizations’ Decisions to Accept Dogs from Distant Locations
Source: Animals (Basel). 2016 Feb 3;6(2):11. doi: 10.3390/ani6020011 (PMC4773738; doi:10.3390/ani6020011)
Supplement: Supplementary File 1 [file animals-06-00011-s001.pdf]

Supplementary Materials: Dogs on the Move: Factors Impacting Animal Rescue Organizations' Decisions to Accept Dogs from Distant Locations

Kaitlyn E. Simmons and Christy L. Hoffman

Dog Transfer Survey Consent Form

You are being asked to participate in a research study conducted by Kaitlyn Simmons under the guidance of Dr. Christy Hoffman from Canisius College. If you have any questions or concerns about the research, please feel free to contact Kaitlyn Simmons at Canisius College by email (simmonsk@my.canisius.edu).

Purpose of the Study

The goal of this study is to learn more about programs between shelter and rescue organizations that are responsible for transferring dogs over long distances (>100 miles). To be eligible to participate in this study, your organization must be involved in receiving dogs from other shelters/rescues as part of a long distance, animal transfer program. Survey participants must be individuals responsible for making decisions regarding which dogs will be transferred into their facility. We request that only one person per organization completes the survey.

Procedures

This survey is expected to take about 25–30 min. It must be completed in one sitting. You cannot come back to complete it.

Potential Benefits to Participants and/or to Society

The findings from this study will be useful to individuals and organizations interested in learning about factors that influence shelters' and rescues' decisions to transport shelter dogs long distances as part of the rehoming process.

Compensation for Participation

Participants can enter to win a $35 Amazon gift card. The contact information collected for the gift card raffle is not linked to any survey responses.

Confidentiality

Any information obtained in connection with this study is confidential. Participants' responses are anonymous. We do not seek to collect any information that would enable us to identify individual participants or the organizations they represent.

Participation and Withdrawal

By completing this survey, you give consent for your data to be used by the researchers. Once you complete the survey and submit it, you will not be able to withdraw from the study because your data are anonymous and thus cannot be identified as yours.

Feedback on the Results of this Study to the Participants

Once the data have been analyzed and the study is completed, you are welcome to view the findings of the study. If you are interested in these, please send a separate email to Kaitlyn Simmons at simmonsk@my.canisius.edu.

Subsequent Use of Data

These data may be used in subsequent studies, in scientific publications and in presentations.

Rights of Research Participants

This study has been approved by the Canisius College Institutional Review Board. If you have any questions regarding your rights as a research participant, you may contact: Michael Dolan at mdolan@canisius.edu

By clicking Next, you are indicating that you are over 18 years of age and are voluntarily consenting to participate in this survey.

Long Distance Dog Transfers

* 1. To your knowledge, has anyone else at your organization completed this survey?

- Yes
- No

* 2. As part of your current position at this organization, are you directly responsible for making (or helping to make) decisions regarding which dogs to transfer into your facility?

- Yes
- No

* 3. Does your organization transfer in dogs from distances greater than 100 miles?

- Yes
- No

* 4. What is your age?

- Under 18
- 18 to 24
- 25 to 34
- 35 to 44
- 45 to 54
- 55 to 64
- 65 or older

* 5. Do you reside in the United States (including U.S. territories)?

- Yes
- No

* 6. What best describes your organization?

- A municipal agency run by the city or county government
- A private agency operating without government contracts or involvement
- A private agency with a government contract to provide animal control services
- Other (please specify)

* 7. How does your organization classify itself?

- Rescue
- Shelter
- Sanctuary
- Other (please specify)

* 8. Which best describes your organization?

- Open access (your organization accepts all dogs surrendered to it by the public)
- Restricted access (your organization places limits on the numbers and/or types of dogs it accepts)

* 9. In what state or U.S. territory is your organization located?

State/Province -- select state --

* 10. Are you male or female?

- Male
- Female

* 11. What best describes your role at this organization?

- Staff Member (paid)
- Volunteer (unpaid)
- Other (please specify)

* 12. What type of region best describes where your shelter is located?

- Rural
- Suburban
- Urban
- Other (please specify)

* 13. Approximately how many dogs enter your organization each year?

* 14. Approximately how many dogs are adopted from your organization each year?

* 15. How many paid staff members does your organization have?

- 1-5
- 6-10
- 11 or more
- All volunteer run (no paid staff)

* 16. What does your organization operate out of?

- A stand-alone building
- A network of foster homes
- Other (please explain)

Please answer all of the following survey questions as they pertain to the long distance (>100 miles) dog transfers that your organization is involved in.

Long Distance Dog Transfers

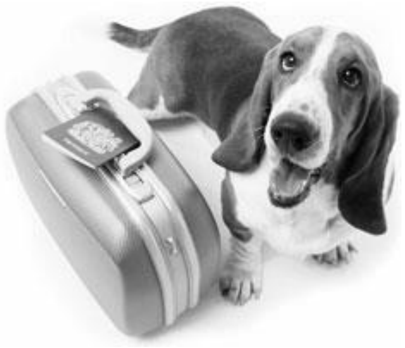

Image credit: http://petpicsdaily.com/pet-advice/travel-with-your-pet/

* 17. Who funds the long distance dog transfers for your organization?

- Your organization
- The organization sending the animal
- Other (please specify)

* 18. On average, how many dogs does your shelter transfer in from distances greater than 100 miles annually?

* 19. Please select the answer choice that best represents how your organization engages in long distance dog transfer programs.

- There are somewhere between 1 and 5 organizations with which we have a relationship and from which we routinely transfer in dogs.
- There are somewhere between 6 and 10 organizations with which we have a relationship and from which we routinely transfer in dogs.
- There are more than 10 organizations with which we have a relationship and from which we routinely transfer in dogs.
- Each time we transfer in dogs, we tend to acquire them from a different organization.
- Other (please explain)

* 20. Are there any shelters/rescues from which your organization has previously transferred in dogs long distance that your organization will no longer work with?

- Yes
- No

Long Distance Dog Transfers

* 21. Please indicate the reason or reasons why your organization chooses not to participate in long distance dog transfers with certain organizations.

- ☐ A dog or dogs that came from this organization had severe behavioral problems.
- ☐ A dog or dogs that came from this organization had severe medical problems
- ☐ The organization did a poor job of representing to my organization a dog's (or dogs') behavioral needs prior to transfer.
- ☐ The organization did a poor job of representing to my organization a dog's (or dogs') medical needs prior to transfer.
- ☐ I (or my organization) felt the transferring organization intentionally deceived us in their effort to relocate a dog (or dogs).
- ☐ The organization does not tend to have dogs that are of interest to prospective adopters in my community.
- ☐ Other (please explain)

* 22. Who transports the dogs from their point of origin to your organization in your long distance transfers (select all that apply)?

- ☐ Paid staff from your organization
- ☐ Volunteers from your organization
- ☐ An independent organization that is paid for their services
- ☐ An independent organization that volunteers their services
- ☐ Staff or volunteers associated with the shelter of origin
- ☐ None of the above (please explain)

* 23. Regarding your organization's long distance transfers, how do the dogs travel to you (select all that apply)?

- ☐ Airplane
- ☐ Car/van
- ☐ Train
- ☐ Other (please specify)

Long Distance Dog Transfers

* 24. Does your organization have a mandatory quarantine period?

- ◯ Yes, but only for dogs transferred from outside the community
- ◯ Yes, for all dogs entering our facility
- ◯ No

* 25. How long is the quarantine period? Please specify in days.

* 26. Who is responsible for carrying out the quarantine?

- ◯ Your organization
- ◯ The organization transferring to you
- ◯ Other (please specify)

* 27. Is it your organization's policy that dogs be spayed/neutered PRIOR TO long distance transfers into your organization?

- ◯ Yes
- ◯ No

* 28. Is it your organization's policy that dogs undergo certain medical treatments (including receiving particular vaccinations) PRIOR TO long distance transfers into your organization?

- ◯ Yes
- ◯ No

* 29. Please describe all mandatory medical treatments that you require dogs to undergo prior to transfer into your facility. Please be specific and include any/all required vaccines.

* 30. Please rate how important each of the following factors is to your organization when deciding which dogs to transfer into your organization from distances greater than 100 miles.

	Not at all important	Not very important	Somewhat important	Very important
Bite history	◯	◯	◯	◯
Photograph(s) of the dog	◯	◯	◯	◯
Description of the dog	◯	◯	◯	◯
Breed	◯	◯	◯	◯
Size	◯	◯	◯	◯
Coat color	◯	◯	◯	◯
Stories that evoke media attention or stir public sentiment	◯	◯	◯	◯
Shelter space or foster home availability at your organization	◯	◯	◯	◯
Shelter space at the location where the dog is currently housed	◯	◯	◯	◯

Long Distance Dog Transfers

* 31. Please rate how important each of the following factors is to your organization when deciding which dogs to transfer into your organization from distances greater than 100 miles.

	Not at all important	Not very important	Somewhat important	Very important
The likelihood the dog will be euthanized at his/her current shelter	○	○	○	○
Public interest	○	○	○	○
Potential for positive community relations	○	○	○	○
Potential for disease transmission	○	○	○	○
Age of the dog	○	○	○	○
Sex of the dog	○	○	○	○
Emotional appeal of the dog	○	○	○	○
The dog already being spayed or neutered	○	○	○	○
Funding	○	○	○	○

* 32. Which is your organization more likely to accept for transfer from distances greater than 100 miles?

○ A purebred dog
○ A mixed breed dog of unknown origin
○ A mix primarily of a purebred dog but without definite purebred origin
○ No opinion

* 33. How likely would your organization be to transport in dogs of the following sizes from distances greater than 100 miles?

	Not at all likely	Unlikely	No opinion	Somewhat likely	Very likely
An extra small dog (under 10 pounds)	○	○	○	○	○
A small breed dog (11-25 pounds)	○	○	○	○	○
A medium dog (26-50 pounds)	○	○	○	○	○
A large dog (51-90 pounds)	○	○	○	○	○
An extra large dog (over 90 pounds)	○	○	○	○	○

* 34. How likely would your organization be to transport in dogs of the following ages from distances of greater than 100 miles?

	Not at all likely	Unlikely	No opinion	Somewhat likely	Very likely
Less than 12 weeks	○	○	○	○	○
3 months to 6 months old	○	○	○	○	○
7 months to 11 months old	○	○	○	○	○
1 year to 2 years old	○	○	○	○	○
3 years to 5 years old	○	○	○	○	○
6 years to 8 years old	○	○	○	○	○
9 years old and older	○	○	○	○	○

Long Distance Dog Transfers

* 35. Are there any breeds or breed mixes that your organization will NOT transfer in from distances of greater than 100 miles?

- Yes
- No

* 36. Please list any breeds or breed mixes that your organization will NOT transfer in from distances of greater than 100 miles (please be as specific as possible).

* 37. What information is necessary to know prior to making long distance transfer decisions for your organization?

* 38. If an organization reaches out to you for help but does not provide all the information your organization requires when making long distance transfer decisions, what do you do?

- Always follow up with the organization and request that they send the required information
- Only follow up with the organization and request they send the required information if the animals appear highly desirable to your organization
- Move on to an organization who sent the appropriate information with their animal transfer request
- Other (please specify)

* 39. How likely would your organization be to transfer in dogs (from distances greater than 100 miles) from the following situations?

	Definitely will not	Unlikely	Likely	Definitely will
Hoarding				
Dog fighting raids				
International transports				
Abusive situations/homes				
Across state lines				
Situations where BSL(breed selective legislation) has left a dog homeless or at risk of being euthanized				
Death of an owner				
Lack of space at a transferring shelter due to disease outbreak				
Puppy mill seizures				
Natural disasters				

Long Distance Dog Transfers

* 40. Do you think that being involved in long distance dog transfer programs positively impacts your organization?

- Yes
- No
- No opinion

* 41. Please answer the following questions regarding your personal opinions on long distance dog transfer programs.

	Strongly Disagree	Disagree	Neither Disagree Nor Agree	Agree	Strongly Agree
I believe that dog transfer programs can help lower euthanasia rates across the nation.					
I participate in dog transfer programs only because my organization does.					
Dog transfer programs are overrated and do not help raise adoption rates or lower euthanasia rates.					
I believe that dog transfer programs help raise publicity for our organization.					
I believe that dog transfer programs, when done correctly, can help combat the pet overpopulation problem.					
I am unsure if dog transfer programs are helpful					
Transferring dogs into our facility makes it harder to adopt out dogs we currently have.					
I believe that dog transfer programs can lead to the spread of animal disease.					
Dog transfer programs are not worth all the effort and time that staff have to put into the logistical details required.					
I believe that dog transfer programs enable shelters to provide their communities with dogs of particular breeds, ages or sizes that individuals would otherwise purchase.					

Long Distance Dog Transfers

* 42. On average, how attractive to your organization's adopters are dogs from long distance transfer programs compared to dogs that come to your organization from local sources?

- Much more attractive to adopters
- More attractive to adopters
- Equally as attractive to adopters
- Less attractive to adopters
- Much less attractive to adopters

* 43. When a dog that was transferred to your organization from long distance transfers is adopted, who keeps the adoption fee?

- Your organization
- The organization who transferred the animal to you
- The adoption fee is divided between my organization and the organization from which the dog originated
- Other (please specify)

44. Optional: Please feel free to include in the box below any additional thoughts you would like to share regarding long distance animal transfers. If you have had particularly good or bad experiences related to long distance animal transfer efforts, please feel free to describe them here.

Thank you for taking the time to complete this survey. Your responses are greatly appreciated. If you would like a copy of the study findings once they become available, please send an e-mail to Kaitlyn Simmons (simmonsk@my.canisius.edu).

45. Optional: To enter a raffle to win a $35 Amazon gift card, please enter your email address below. Your address is confidential and is in no way connected to your responses on this survey.

Email Address
